# Investigation of the Cellular Pharmacological Mechanism and Clinical Evidence of the Multi-Herbal Antiarrhythmic Chinese Medicine Xin Su Ning

**DOI:** 10.3389/fphar.2020.00600

**Published:** 2020-05-06

**Authors:** Yu-ling Ma, Rou-Mu Hu, Xinchun Yang, Taiyi Wang, Penelope J. Noble, Robert Wilkins, Clive Ellory, Carolyn Carr, Denis Noble, Jiefu Yang, Weixing Lu, Junhua Zhang, Hongde Hu, Xiaomei Guo, Min Chen, Yang Wu, Meng Wei, Jingyuan Mao, Xiaochang Ma, Ling Qin, Huanlin Wu, Feng Lu, Ying Cao, Sheng Gao, Wanli Gu

**Affiliations:** ^1^Oxford Chinese Medicine Research Centre & Department of Physiology, Anatomy and Genetics, University of Oxford, Oxford, United Kingdom; ^2^Heart Center & Beijing Key Laboratory of Hypertension, Beijing Chaoyang Hospital, Capital Medical University, Beijing, China; ^3^Department of Cardiology, Beijing Hospital, National Center of Gerontology, Beijing, China; ^4^Department of Cardiology, Beijing University of Chinese Medicine Third Affiliated Hospital, Beijing, China; ^5^Institute of Traditional Chinese Medicine, Tianjin University of Traditional Chinese Medicine, Tianjin, China; ^6^Department of Cardiovascular Diseases, West China Hospital, School of Clinic Medicine, Sichuan University, Chengdu, China; ^7^Department of Cardiology, Tongji Hospital, Tongji Medical College, Huazhong University of Science and Technology, Wuhan, China; ^8^Geriatrics Department, Affiliated Hospital of Liaoning University of Traditional Chinese Medicine, Shenyang, China; ^9^Clinical Departments of Cardiology, Dongfang Hospital Beijing University of Chinese Medicine, Beijing, China; ^10^Department of Cardiology, Shanghai Jiao Tong University Affiliated Sixth People’s Hospital, Shanghai, China; ^11^Department of Cardiology, First Teaching Hospital of Tianjin University of Traditional Chinese Medicine, Tianjin, China; ^12^Department of Cardiology, Xiyuan Hospital CACMS, Beijing, China; ^13^Department of Cardiology, the First Hospital of Jilin University, Changchun, China; ^14^Department of Cardiology, Guangdong Provincial Hospital of Chinese Medicine, Guangzhou, China; ^15^Department of Cardiology, the Affiliated Hospital of Shandong University of Traditional Chinese Medicine, Jinan, China; ^16^Department of Cardiology, Hengyang Hospital of Traditional Chinese Medicine, Hengyang, China; ^17^Department of Cardiology, Tianjin Hospital of ITCWM Nankai Hospital, Tianjin, China; ^18^Department of Traditional Chinese Medicine, Liaocheng People’s Hospital, Liaocheng, China

**Keywords:** Xin Su Ning, traditional Chinese medicine, multicomponent antiarrhythmic medicine, cellular electrophysiology, premature ventricular contraction

## Abstract

Xin Su Ning (XSN), a China patented and certified multi-herbal medicine, has been available in China since 2005 for treating cardiac ventricular arrhythmia including arrhythmia induced by ischemic heart diseases and viral myocarditis, without adverse reactions being reported. It is vitally important to discover pharmacologically how XSN as a multicomponent medicine exerts its clinical efficacy, and whether the therapeutic effect of XSN can be verified by standard clinical trial studies. In this paper we report our discoveries in a cellular electrophysiological study and in a three-armed, randomized, double-blind, placebo-controlled, parallel-group, multicenter trial. Conventional electrophysiological techniques were used to study the cellular antiarrhythmic mechanism of XSN. Data was then modeled with computational simulation of human action potential (AP) of the cardiac ventricular myocytes. The clinical trial was conducted with a total of 861 eligible participants randomly assigned in a ratio of 2:2:1 to receive XSN, mexiletine, or the placebo for 4 weeks. The primary and secondary endpoint was the change of premature ventricular contraction (PVC) counts and PVC-related symptoms, respectively. This trial was registered in the Chinese Clinical Trial Register Center (ChiCTR-TRC-14004180). We found that XSN prolonged AP duration of the ventricular myocytes in a dose-dependent, reversible manner and blocked potassium channels. Patients in XSN group exhibited significant total effective responses in the reduction of PVCs compared to those in the placebo group (65.85% vs. 27.27%, P < 0.0001). No severe adverse effects attributable to XSN were observed. In conclusion, XSN is an effective multicomponent antiarrhythmic medicine to treat PVC without adverse effect in patients, which is convincingly supported by its class I & III pharmacological antiarrhythmic mechanism of blocking hERG potassium channels and hNaV1.5 sodium channel reported in our earlier publication and prolongs AP duration both in ventricular myocytes and with computational simulation of human AP presented in this report.

## Introduction

Although much research has been carried out in the attempt to gain better understanding of cardiac arrhythmia and in searching for effective and safe drugs to treat this common cardiac condition, progress is not very encouraging due to the multifactor and dynamic nature of the disease’s causes and its development (Weiss et al., 2015). Targeting ion channels or cellular electrophysiological properties as new drug development strategy has developed sophistically in the last decades, however using these methods, the drugs discovered in this scheme are far from satisfactory (Frommeyer and Eckardt, 2016).

Xin Su Ning (XSN) is a multi-herbal medicine patented and produced in China, which is sold in China since 2005 for treating cardiac ventricular arrhythmia, especially arrhythmias induced by cardiac ischemia and viral myocarditis. XSN is comprised of 11 herbs: Coptidis Rhizoma (Huanglian, *Coptis chinensis* Franch.), Pinelliae Rhizoma (Banxia, *Pinellia ternata* (Thunb.) Makino), Poria (Fuling, *Poria cocos* (Schw.) Wolf), Aurantii Fructus Immaturus (Zhishi, *Citrus aurantium* L.), Dichroae Radix (Changshan, *Dichroa febrifuga* Lour.), Nelumbinis Plumula (Lianzixin, *Nelumbo nucifera* Gaertn.), Sophorae Flavescentis Radix (Kushen, *Sophora flavescens* Ait.), Artemisiae Annuae Herba (Qinghao, *Artemisia annua* L.), Ginseng Radix et Rhizoma (Renshen, *Panax ginseng* C. A. Mey.), Ophiopogonis Radix (Maidong, *Ophiopogon japonicus* (L. f) Ker Gawl.), and Nardostachyos Radix et Rhizoma (Gancao, *Glycyrrhiza uralensis* Fisch.). The pre-licensing pharmacological studies in China showed that XSN significantly suppresses cardiac arrhythmia induced by chemical reagents: matrine, CaCl_2_, chloroform, and isoproterenol; and in cardiac ischemia-induced arrhythmia, XSN significantly delayed the onset of ventricular arrhythmia and shortened the time of arrhythmia. The pre-licensing toxicity study showed that XSN did not induce any abnormal changes to blood and urine, and application of XSN did not cause any toxic reactions in the heart, the liver, and kidney. We have recently reported that XSN blocks hERG potassium and hNav1.5 ion channels (Wang et al., 2019b), which implies that XSN can be categorized as a class I & III antiarrhythmic medicine. In order to evaluate scientifically the efficacy rigorously, in 2015 after 10 years of clinical use, a three-armed, randomized, double-blind, placebo-controlled, parallel-group, multicenter trial was launched with 861 eligible patients randomly assigned.

We report here the cellular electrophysiological mechanism of XSN, which would help to explain how XSN exert its antiarrhythmic action in patients. In supporting the mechanistic discovery we also report the results of the clinical trial. Both laboratory and clinical research data are not only mutually supporting each other, but also providing strong scientific evidences regarding the widely accepted clinical efficacy of XSN in treating cardiac arrhythmias since 2005.

The cellular electrophysiological study was conducted using the conventional electrophysiological techniques (Ma et al., 2006) that have been used for decades to identify/classify antiarrhythmic chemical drugs and compounds (Rosen, 1988). The effect of XSN on the action potential (AP) and its corresponding ion channels of the ventricular myocytes were studied aiming to clarify the cellular antiarrhythmic mechanism of XSN, which in turn would provide mechanistic explanation for the clinical efficacy of XSN.

We then report the results of the anti-premature ventricular contraction (PVC) clinical evidence of XSN. PVCs, also known as premature ventricular complexes, are early depolarization of the myocardium originating in the ventricle due to increased automaticity, triggered activity, or reentry. PVCs are commonly encountered in apparently healthy individuals with a reported incidence of approximately 1% on surface 12-lead electrocardiogram (ECG) (Saurav et al., 2015). PVCs are seen in 40% of routine 24-h ambulatory ECG recordings and in 75% of 48-h recordings among the general population referred for ambulatory ECG monitoring. Prevalence increases with age and with less than 1% of ambulatory ECG recordings demonstrating PVCs in those younger than 11 years of age compared with more than 69% in those aged older than 75 years (Eugenio, 2015).

Despite PVCs being considered as benign in the absence of structural heart disease, some of the recent data on PVCs casts serious doubt on this hitherto-perceived notion. PVCs might produce high PVC burden in select individuals, contribute to left ventricular (LV) dysfunction and dilation, and hemodynamic deterioration (Winkens et al., 2014). Frequent PVCs cause severe symptoms such as dyspnea, chest pain, dizziness, and palpitation and may even be incapacitating in some patients (Cantillon, 2013; Giles and Green, 2013). Furthermore, more and more recent studies have shown that frequent PVCs in patients with established heart disease are associated with increased risk of cardiac mortality (Qureshi et al., 2014; Lee et al., 2015). Meta-analysis of premature ventricular complexes demonstrated that frequent PVCs are associated with a substantial increase in the risk for sudden cardiac death and total cardiac death findings from observational studies in general populations (Ataklte et al., 2013). Some studies have also shown that symptomatic PVCs, and especially PVCs elicited during exercise, even in the absence of identifiable heart disease, are correlated with an increased risk of total mortality (Frolkis et al., 2003). Thus, the prevention and management of PVCs are major public health challenges.

Evidence-based management of frequent PVCs usually includes pharmacological treatment and catheter ablation therapy for symptomatic patients who failed to respond to medical therapy (Adams et al., 2012). Conventional anti-arrhythmic drugs (AADs) such as β blockers or class I or III anti-arrhythmic agents are the first-line recommendation for patients with symptoms that can be attributed to frequent PVCs. However, the long-term use of single chemical AADs may be suboptimal in the treatment of frequent PVCs either due to their significantly low efficacy or proarrhythmic effects (Kowey and Yan, 2005). Even with the advance of catheter ablation for ventricular tachycardia, AADs remain an important tool for treating arrhythmias. Therefore, seeking for a new effective and safe anti-arrhythmic medicine/method is an important subject of PVCs treatment.

Complementary and alternative medicine (CAM) therapy has a long history of use in China to treat chronic diseases (Xiong et al., 2013). Traditional Chinese Medicine (TCM), as one of the CAM therapies, has anti-arrhythmic properties similar to prescription of AADs and may have low toxicity and less adverse clinical events with respect to multiple targets, significant efficacy, and safety (Brenyo and Aktas, 2014; Hua et al., 2015; Liu et al., 2015).

Previous studies have supported the ability of XSN to suppress and prevent cardiac arrhythmias, including atrial, and ventricular arrhythmias. However, the clinical efficacy and potential herb-drug related safety regarding XSN used in humans has yet to be studied. Therefore, to further confirm the efficacy of XSN observed in experimental results, a three-armed, randomized, double-blind, placebo-controlled, parallel-group, multicenter trial was designed to systematically evaluate the efficacy and safety of XSN in the treatment of patients with PVCs.

## Materials and Methods

### Medicine and Chemicals

The experimental preparation of XSN was provided by Shaanxi Momentum Pharmaceutical Co., Ltd. (Shaanxi, China) as an frozen dried powder of the extract of the 11 herbs described above (detailed preparation procedures is described in the **Supplementary Document**). The chemical composition of XSN was studied and has been reported recently (Guo et al., 2018); using the ultra-high-pressure liquid chromatography coupled with linear ion trap-Orbitrap tandem mass spectrometry (UHPLC-LTQ-Orbitrap) the fingerprint of the chemical composition of XSN is shown in the supplement as **Supplementary Figure S1** with the authors’ permission, 41 components were identified as candidate bioactive components (Guo et al., 2018). All other chemicals were purchased from Sigma-Aldrich (Gillingham, Dorset, UK) and used as supplied unless stated.

### Cellular Electrophysiological Study

#### Cell Isolation

Cardiac myocytes were isolated from the hearts of adult male Wistar rats (250–300 g) in accordance with UK Home Office regulations (Schedule I of A [SP] A 1986), approved by national and University ethics committees. Rats were anesthetized with isoflurane and the hearts removed rapidly and perfused with Krebs Hensleit Bicarbonate (KHB) containing in mM: NaCl, 118.1; KCl, 3.0; CaCl_2_, 1.8; MgSO_4_,1.2; KH_2_PO_4_, 1.0; NaHCO_3_, 27.3; glucose, 10.0; and pyruvic acid, 2.5, pH 7.4. The perfusion rate was set at 5 ml/min at 37°C equilibrated with 95% O_2_—5% CO_2_, and run continuously for 15 min, followed by a low Ca^2+^ KHB containing in mM: NaCl, 105.1; 3.0; CaCl_2_, 0.01; MgSO_4_, 1.2; KH_2_PO_4_, 1.0; NaHCO_3_, 20.0; Glucose, 10.0; pyruvic acid, 5.0; taurine, 10.0; and mannitol, 5.0, pH 7.4, for an additional 10 min. The perfusion was then switched to a recirculating KHB with low Ca^2+^ containing Liberase Blendzyme 3 (0.03 mg/ml, 40 ml total) for 50 min. The ventricle was minced and passed through four layers of gauze, and the solution with the cells was centrifuged at 50 g for 2 min, and the supernatant aspirated. The cells were finally resuspended in low Ca^2+^ solution and were kept at 4°C until use.

#### Electrophysiological Techniques and Data Analysis

Myocytes were continuously superfused with recording solution in a recording chamber mounted on an inverted microscope at room temperature (22–24**°**C) (Ma et al., 2006). APs were recorded using the whole-cell configuration of the patch-clamp technique in current clamp mode with an Axon 200B patch-clamp amplifier. Recording pipettes, made from borosilicate glass, had resistances of between 1.5 and 3 M when filled with the pipette solution containing in mM: KCl, 120.0; CaCl_2_, 1.0; MgCl_2_, 2.0; ATP_Na_, 3.0; HEPES, 10.0; and EGTA, 11.0, pH 7.2 for AP recordings, and the extracellular solution containing in mM: NaCl, 112.1; KCl, 5.4; CaCl_2_, 1.0; MgCl_2_, 1.0; KH_2_PO_4_, 1.0; NaHCO_3_, 24.0; and glucose, 10.0; pH 7.4. The whole-cell I_K_ current was recorded in the voltage clamp mode of the amplifier, and the extracellular solution containing in mM: choline Cl, 112.1; KCl, 5.4; CaCl_2_, 1.0; CdCl_2_, 0.1; MgCl_2_, 1.0; KH_2_PO_4_, HEPES, 5.0; NaHCO_3_, 24.0; and glucose, 10.0; pH 7.4. Current and voltage commands were generated with pCLAMP data acquisition (Version 10, Axon Instruments, USA). Data analysis was carried out using pCLAMP (V.10) and Origin (Microcal software, Inc. Northampton, MA) software. The cellular experimental XSN was prepared by standard extraction of the 11 herbs of XSN, after various filtrations a purified solution of XSN was freeze-dried to a powder preparation (by Momentum Pharmaceutical Co. Ltd.), which we used for this study. XSN at various concentrations was dissolved in extracellular solution. The control recordings were taken at least 3 **min** after the AP had stabilized. The data are presented as the mean value and its standard error (mean SE). The differences between control levels and the changes caused by XSN application were tested using Student**’**s t-test.

### Clinical Study

#### Patients Eligibility and Exclusion Criteria

This study was a randomized, double-blind, placebo and positive drug-controlled, multicenter study. Patients with the age of 18–75 years accompanied by PVCs [>3,000 beats/d and <30,000 beats/d as recorded by Ambulatory Electrocardiograph (AECG)] who were willing and able to provide written consent were enrolled to the study. All the eligible patients were required to discontinue other AADs for at least five half-lives or TCM related to cardiovascular diseases for 2 weeks prior to enrollment. Both men and women were included. Exclusion criteria included the following: (1) severe patients needing other AADs; (2) acute myocardial infarction, acute heart failure, systemic diseases such as anemia, hyperthyroidism, PVCs due to digitalism, electrolyte disturbances or other reversible factors, left ventricular ejection fraction (LVEF) < 45%; (3) pregnant and lactating women, severe mental illness; (4) disabled patients; (5) primary hepatic, renal or hematopoietic system disease, alanine aminotransferase (ALT), aspartate aminotransferase (AST) levels > twofold of the upper normal limit or serum creatinine levels higher than the upper normal limit; (6) patients who have received percutaneous coronary intervention or coronary artery bypass grafting in recent three months; (7) hypertension class III or highly risky with poor blood pressure control; (8) a suspicion of allergies to the study drugs; (9) participation in other clinical trials within 3 months prior to enrollment.

The study protocol was reviewed and approved by the institutional review boards of each participating center, and the study was conducted in accordance with the principles of the *Good Clinical Practice Guidelines* and the *Declaration of Helsinki*. All patients provided written informed consent. The study was registered in the Chinese Clinical Trial Register Center (ChiCTR-TRC-14004180).

#### Study Design

**Figure 1:**
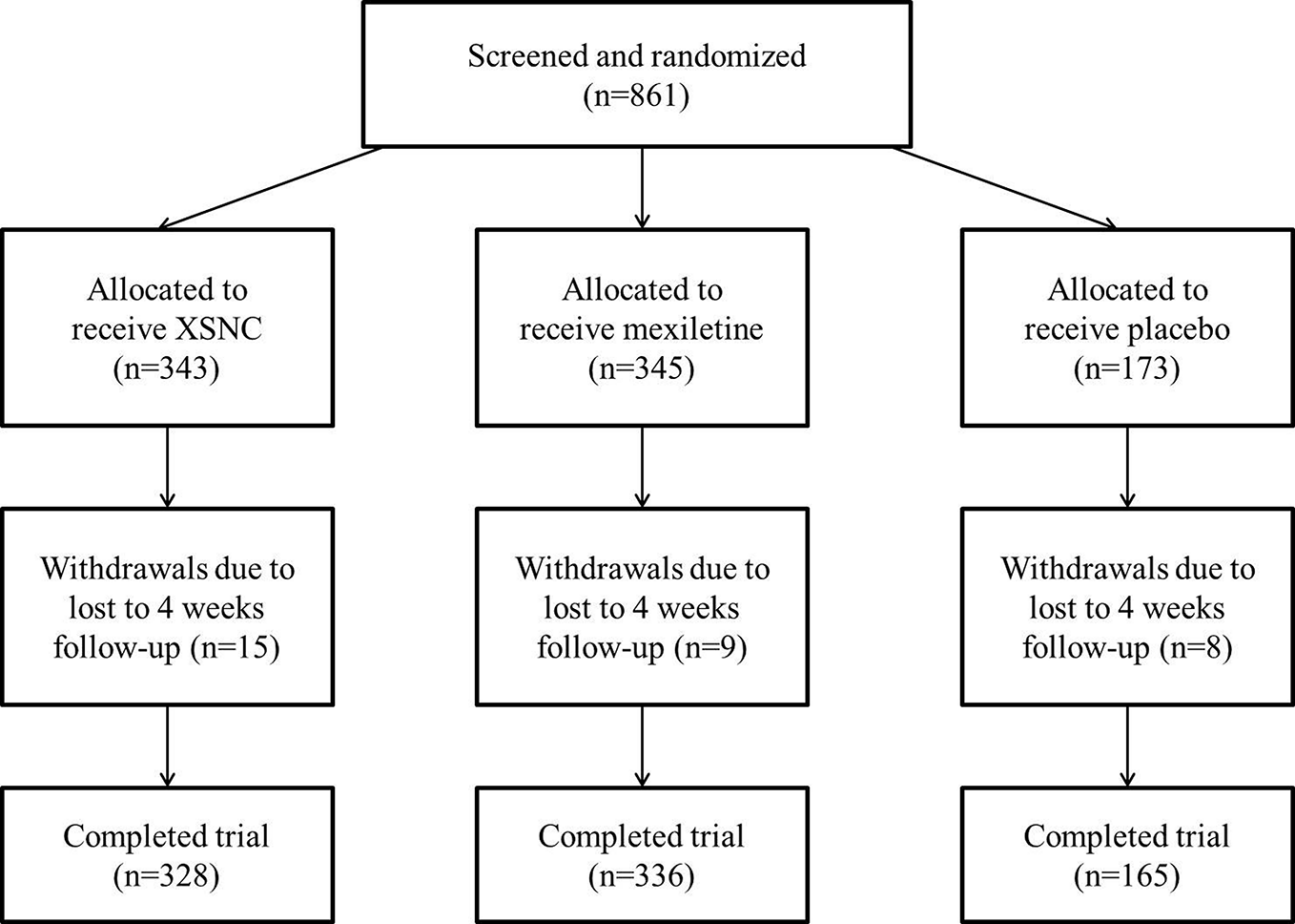
Flowchart detailing enrollment and follow-up.

This study was designed as a three-armed, randomized, double-blind, placebo and positive drug parallel-controlled, multicenter study in patients with PVCs. The present study consisted of a run-in period prior to randomization and a treatment period of 4 weeks. All of the patients had undergone a medical history review, physical examinations, laboratory measurements (including serum glucose, electrolyte, ALT, AST, blood urea nitrogen (BUN), and creatinine), 12-lead ECG and AECG screening, and echocardiography examination before enrollment. The eligible patients were then randomly assigned (2:2:1), *via* the computer-generated block randomization prepared in advance, to XSN (XSN four capsules, 0.48 g per capsule plus simulated mexiletine two pills), mexiletine (mexiletine two pills, 50 mg per pill plus simulated XSN four capsules), or placebo groups (simulated XSN four capsules plus simulated mexiletine two pills). Each participant took the treatments three times per day for 4 weeks. Symptoms, physical examinations, vital signs, 12-lead ECG, AECG, echocardiography, and laboratory examinations were performed at baseline and at the final visit. The efficacy and safety of XSN were assessed after 4 weeks of treatment. The flowchart of study enrollment and follow-up was presented in **Figure 1**.

#### Randomization and Blinding

Stratiﬁed and blocked randomization was used according to different centers, and the 39 centers were divided into 39 strata. Patients in each center were further divided into trial, positive drug, and placebo groups using blocked randomization. Based on the results of the sample size estimated, a random number from 1 to 900 was generated by SAS software (version 9.13, SAS Institute Inc., USA), and the subjects were randomly assigned by each center. The results of the randomized allocation were sent by the distribution systems of the responsible department to each research center. Each patient’s random number and his/her drug number were same throughout the trial. The placebos were identical to XSN or mexiletine in appearance, size, weight, color, and taste and were packaged in identical boxes and given to patients by research nurses. The investigators, laboratory personnel, statistical processors, and patients enrolled in this trial were blinded to the study group allocation.

#### Outcome Measures

The primary efficacy variable was evaluated as the relative percent reduction of the overall frequency of PVCs from baseline to 4 weeks after treatment detected by 24-h AECG and was defined as the formula: (the number of PVCs during baseline week − the number of PVCs during the last week)/(the number of PVCs during baseline week) × 100. The definitions of the therapeutic effects of XSN on PVCs were as follows: (1) significantly effective response (SER) – the number of PVCs after treatment decreased by 90% or more compared with baseline; (2) effective response (ER) – the number of PVCs after treatment decreased by 50–90% compared with baseline; and (3) no ER (NER) – the number of PVCs after treatment decreased by <50%, no change, or the number of PVCs increased compared with baseline. The total effective rate (TER) of XSN on PVCs was calculated as the combined endpoints of the SER and ER.

The secondary efficacy variable included changes in PVC-related symptom scores from baseline to 4 weeks after treatment. The symptoms used for this assessment included palpitation and chest discomfort. Specific doctors performed the follow-up and finished PVC-related symptoms score assessments each weekend. The frequency and intensity of the symptoms were rated as none, mild, moderate, or severe, which corresponded to symptom scores of 0, 1, 2, and 3 points, respectively.

#### Safety Evaluation

The safety analysis included all randomized patients who received ≥1 dose of XSN. All patients underwent weekly follow-up safety assessments during the study. Vital signs, routine blood tests, liver function and kidney function tests, electrolyte tests, and ECGs both at baseline and at the end of treatment were examined. Adverse events were monitored and determined by the investigator during follow-up using the criteria of definitely, probably, possibly, probably not, and definitely not attributable to XSN. Premature termination of the trial was considered if the patient had a major adverse event or significant worsening of PVC-related symptoms.

#### Sample Size

The sample size was calculated based on the following method. This is a three-armed RCT study. The XSN group vs. mexiletine group was designed as noninferiority and the XSN group vs. placebo group was designed as superiority. The TERs after treatment with mexiletine, XSN, and the placebo for the total number of PVCs were assumed to be 70.5%, 71.0%, and 30.0% respectively. The superiority margin is 25% and the noninferiority margin is with a type I error rate of alpha = 0.025 and a power of 80% (type II error rate of beta = 0.2), and thus, the required sample size for XSN group was 298 at least. According to 2:2:1 design, the sample sizes for the XSN, mexiletine, and placebo groups were 300, 300, and 150 respectively. Accounting for a dropout rate of 20% of the patients after randomization, a total sample size of 900 was required (360 in the XSN group, 360 in the mexiletine group, and 180 in the placebo group).

#### Statistical Analysis

The baseline data from patients who underwent randomization were analyzed as the full analysis set (FAS). The per-protocol set (PPS) included all patients in the FAS who complied with the protocol and completed the study through the final visit. The safety set (SS) included all the participants who received treatment and safety follow-up for at least one time. The analysis of the primary and second efficacy endpoints were performed in both FAS and PPS. The safety parameters were performed in SS. Continuous data were presented as the mean ± standard deviation (SD), and dichotomous data were presented as numbers and percentages. Comparisons of the data between patient groups were performed using independent sample *t*-tests for continuous data. Paired sample *t*-tests were used to compare the pre- and post-treatment data from each group. The Fisher exact test or the Wilcoxon rank sum test was used to compare binary variables or ranked data. Ninety-five percent confidence interval (*CI*, two-sided) was presented as appropriate. All statistical analyses were performed with SAS software. A *P* < 0.05 was considered as statistically significant, and all statistical tests were two-sided.

## Results

### Cellular Electrophysiology

#### The Effect of XSN on the **AP** of Cardiac Ventricular Myocytes

**Figure 2:**
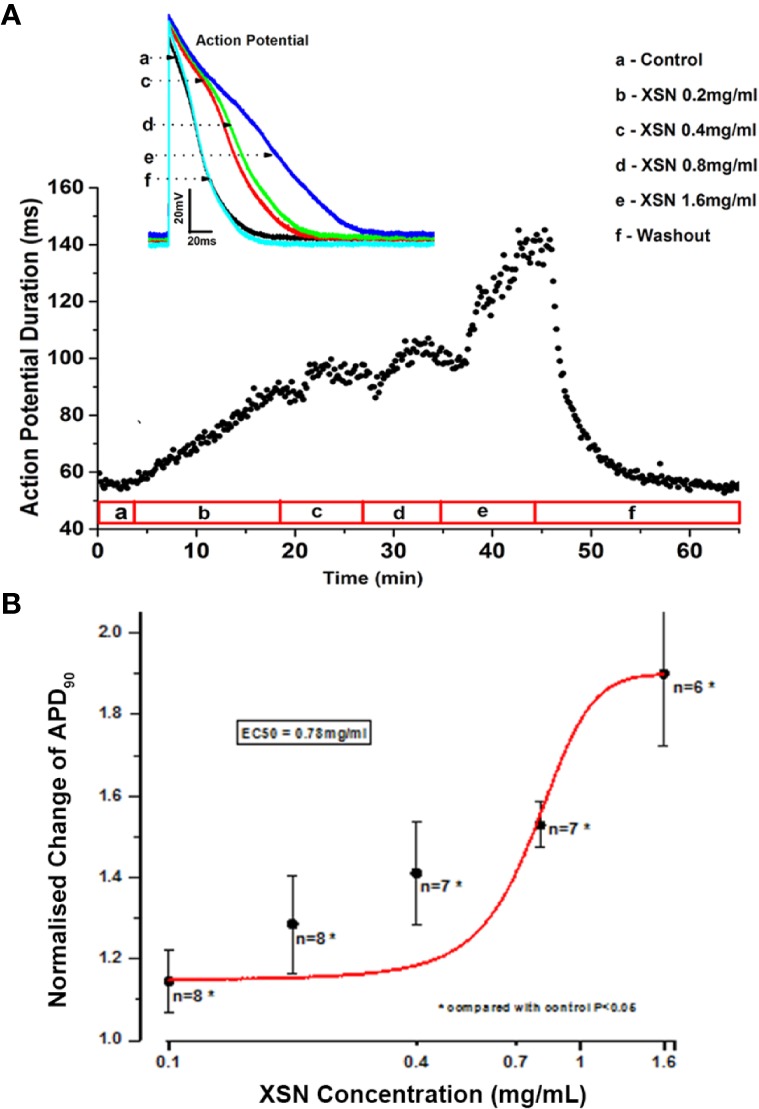
The effect of Xin Su Ning (XSN) on the action potential (AP) of cardiac ventricular myocyte. XSN prolonged APD in a dose-dependent manner. **(A)** APD_90_ data points, extracted from every AP recorded through the entire experiment, plotting against the time with XSN applications at various concentrations indicated in the bar below the plot and the superimposed AP traces recorded at control and in the presence of XSN at different concentrations as the keys indicate. **(B)** The dose-response curve of XSN on APD90 at concentrations ranging from 0.2 to 1.6 mg/ml, with an approximate EC_50_ as 0.78 mg/ml.

The application of XSN at various concentrations to the isolated cardiac ventricular myocytes produced a concentration dependent prolongation of the AP duration (APD) with an EC_50_ of 0.78 mg/ml, which was reversible upon washout of the medicine. **Figure 2A** shows the superimposed AP traces (A) recorded at control, the presence of different concentrations and at washout of XSN as the keys indicated, and the peak current values recorded under different experimental conditions are plotted below against time as the keys indicated. **Figure 2B** is a dose-response curve of XSN with normalized (to the control as 1) APD prolongation against the concentration of XSN; at as low as 0.4 mg/ml, XSN significantly prolonged APD: 0.4 mg/ml, 1.286 ± 0.119 (n=8, P < 0.05); 0.8 mg/ml, 1.463 ± 0.061 (n=9, P < 0.05); and 1.6 mg/ml, 1.803 ± 0.177 (n=7, P < 0.05). The curve fit of the data produced an EC_50_ of 0.78 mg/ml.

#### The Effect of XSN on the Whole-Cell Potassium Current of the Cardiac Ventricular Myocytes

**Figure 3:**
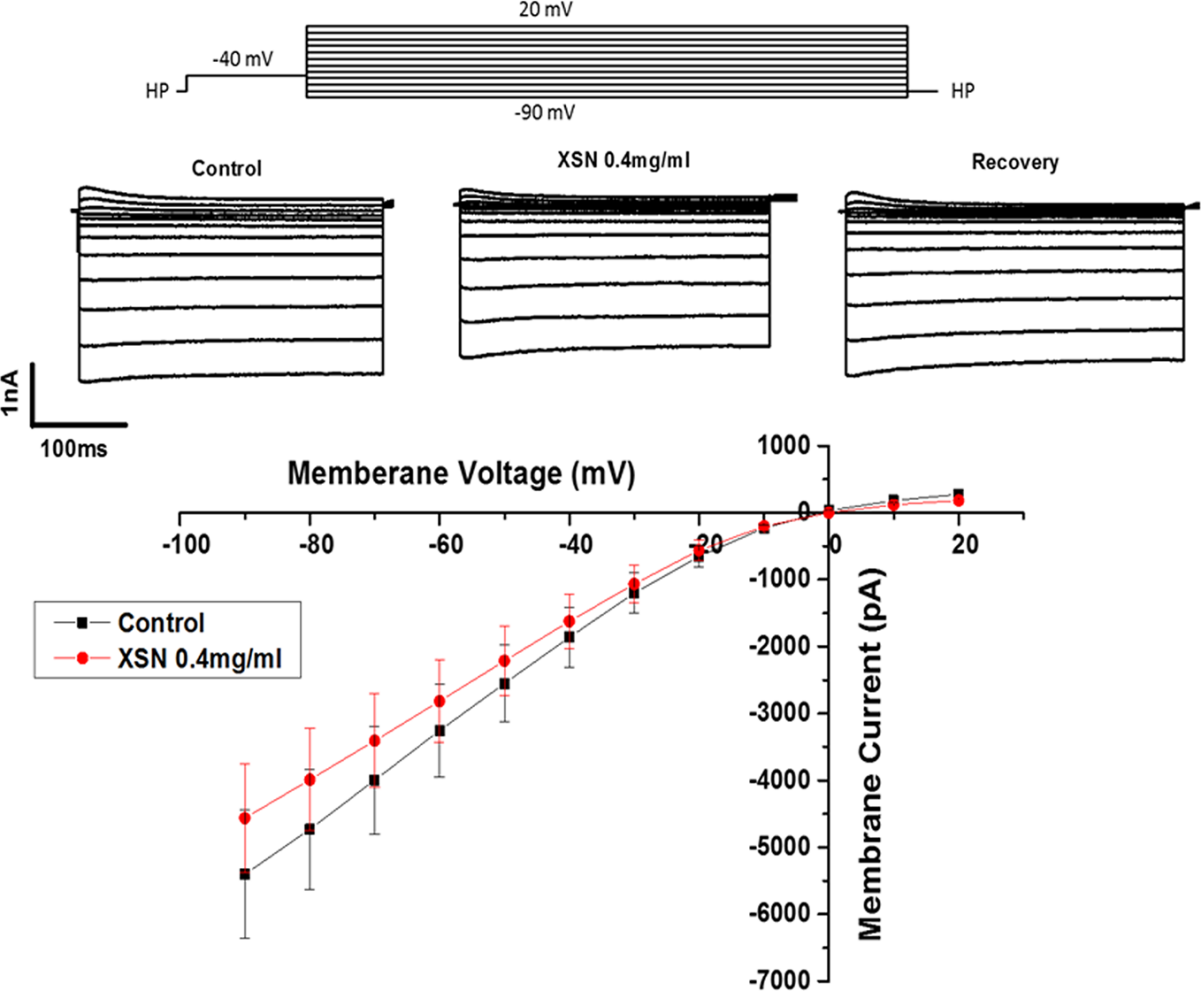
The effect of Xin Su Ning (XSN) on the whole-cell I_k_ of the cardiac ventricular myocytes. The upper panel shows the voltage clamp protocol and the current traces recorded at control, in the presence of XSN and the recovery as indicated above the current traces. The lower panel shows the current–voltage relationship plots at control and in the presence of XSN and the data presented is n=7.

XSN prolonged APD implies that it may block potassium currents to cause the delay of repolarization of the AP. This possibility was tested using a conventional method of recording I_k_ in the isolated cardiac myocytes. **Figure 3** shows the steady-state activation of I_k_ in the absence and presence of XSN, the currents were elicited by applying a series test pulses of 300 ms with potential range from 20 to −90 mV. The results shows that XSN at 0.4 mg/ml suppressed I_K_ and the effect was reversible. This experiment was repeated in seven cells (n=7). The result mechanistically supports the APD propagation property of XSN.

#### The Computational Simulation of the Experimental Data

**Figure 4:**
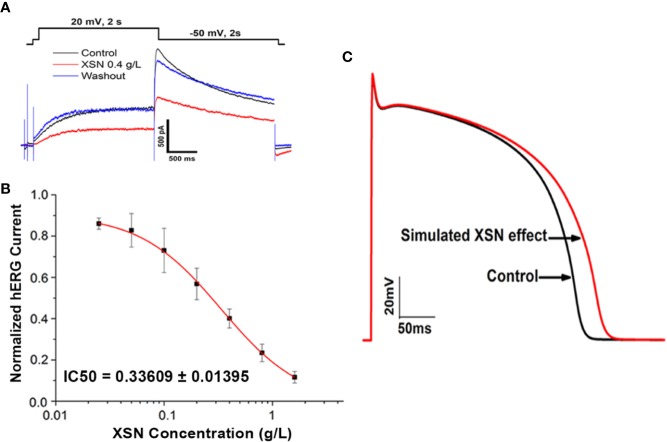
The computational simulation of the human AP. **(A, B)** Data extracted from our previous publication (Wang et al., 2019b); Xin Su Ning (XSN) blocked human hERG channels in a dose-dependent manner with an IC_50_ of 0.34 mg/ml. **(C)** The simulated human AP traces of control and in the presence of 0.4 mg/ml XSN as the keys indicated.

As we reported previously that XSN blocked human hERG channels in a dose-dependent manner with an IC_50_ of 0.34 mg/ml (Wang et al., 2019b); the data were applied to computational simulation of human AP. The result presents a clear class III antiarrhythmic feature of APD prolongation by XSN as shown in **Figure 4**, which also agrees with the results shown in **Figure 2** that XSN prolongs the APD of isolated cardiac myocytes. **Figures 4A, B** are the data extracted from our previous publication (Wang et al., 2019b). **Figure 4C** is the simulated human AP traces of control and in the presence of 0.4 mg/ml XSN as the keys indicated.

#### The Comparison of XSN With One of Its Single Compound Liensinine

**Figure 5:**
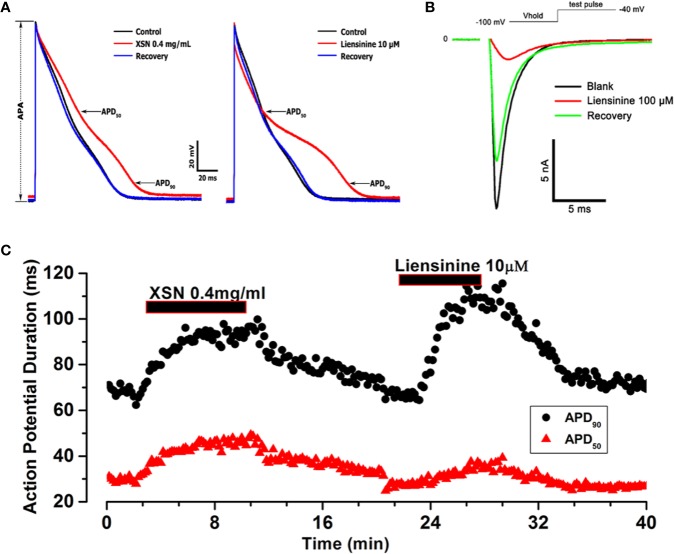
The effect of Xin Su Ning (XSN) and liensinine on action potential (AP) duration. **(A)** The effect of XSN at concentration of 0.4 mg/ml and the effect of liensinine at concentration of 10 µM on AP. **(B)** The effect of Liensinine at 10 µM on I_Na_, and the voltage clamp protocol is shown above the current traces. **(C)** The APD_90_ & APD_50_ plots against time with XSN and Liensinine applications indicated in the keys. The APs and data points in panel **(A–C)** were recorded in one myocyte in a continuous experiment.

There are more than hundred known compounds in XSN; it is the multicomponent medicine that suppresses cardiac arrhythmia effectively in patients. Following conventional thinking, the first question asked would be which compound (s) in XSN induced the prolongation of APD? To try to answer the question we tested a few compounds of XSN, here we present the results on liensinine, extracted from one of the 11 herbs formulated XSN, Nelumbinis Plumula. **Figure 5B** shows that liensinine blocked the voltage-gated fast I_Na_ and significantly prolonged APD with a different profile from XSN (**Figure 5A**). **Figure 5C** shows that XSN at 0.4 mg/ml and liensinine at 10 µM both significantly prolonged APD_90_, However liensinine did not prolong APD_50_ in contrast with XSN: Liensinine at 10 µM prolonged APD_90_ (1.350 ± 0.04, n=4, P < 0.05) without affecting APD_50,_ 0.96 ± 0.03 (n=4, P > 0.05); XSN at 0.4 mg/ml significantly prolonged APD_90_ and APD_50_, 1.22 ± 0.03 (n=5, P < 0.05) and 1.34 ± 0.06 (n=5, P < 0.05), implying that a single compound could not produce the multicomponent action of XSN.

### Clinical Study

#### Patient Characteristics

From April 2014 to January 2016, 861 patients from 39 hospitals across the mainland of China were enrolled in this study and were randomly assigned to receive XSN, mexiletine, or the placebo. Of the 861 patients enrolled, 343 patients were randomized to the XSN group, 345 patients were randomized to the mexiletine group, and 173 patients were randomized to the placebo group. Fifteen patients in the XSN group, nine patients in the mexiletine group, and eight patients in the placebo group were lost to follow up. As a result, a total of 829 patients (328 in the XSN group, 336 in the mexiletine group, and 165 in the placebo group) completed the study. The baseline characteristics of the 829 patients are shown in **Table 1**. The XSN group, mexiletine group and the placebo group were comparable with respect to demographic characteristics, clinical manifestations, AECG parameters, and medical treatments. Of the 861 patients enrolled and randomized, 74 patients due to loss to follow-up or noncompliance with the study protocol. The dropout rate by trial arm was 34 cases in the XSN group (dropout rate: 9.91%), 21 cases in the mexiletine group (dropout rate: 6.09%), and 19 cases in the placebo group (dropout rate: 10.98%). As a result, a total of 779 (90.48%) patients (307 in the XSN group, 320 in the mexiletine group, and 173 in the placebo group) completed the 4-week follow-up and were included in further efficacy analyses.

**Table 1:** Baseline characteristics of the study population.

Items	XSN group (n = 328)	Mexiletine group (n = 336)	Placebo group (n = 165)	P
Age, years	53.50 ± 13.09	52.12 ± 13.62	53.86 ± 13.21	0.3193
Male	121 (39.41)	151 (44.94)	77 (44.67)	0.3132
Height, cm	165.54 (6.93)	166.14 (7.27)	166.01 (7.13)	0.3481
Weight, kg	66.05 (9.88)	66.81 (11.45)	66.03 (10.41)	0.7145
Systolic blood pressure	125.32 (10.86)	125.09 (11.94)	125.13 (11.32)	0.8532
Diastolic blood pressure	77.42 (7.63)	77.64 (7.63)	77.59 (8.34)	0.9948
Symptoms				
palpitation	281 (91.53)	299 (93.44)	148 (97.37)	0.4184
Chest discomfort	267 (86.97)	283 (88.44)	138 (80.79)	0.8606
PVCs, beat	9250.56 (4180.00,11589.50)	8983.23 (4184.50,11593.00)	9129.63 (4091.00,11780.00)	0.5886
PACs, beat	236.48 (0.00,31.00)	233.78 (0.00,33.00)	165 (0.00,34.00)	0.8253

PVCs, premature ventricular contractions; PACs, premature atrial contractions; XSN, Xin Su Ning.

#### Efficacy Analysis

**Figure 6:**
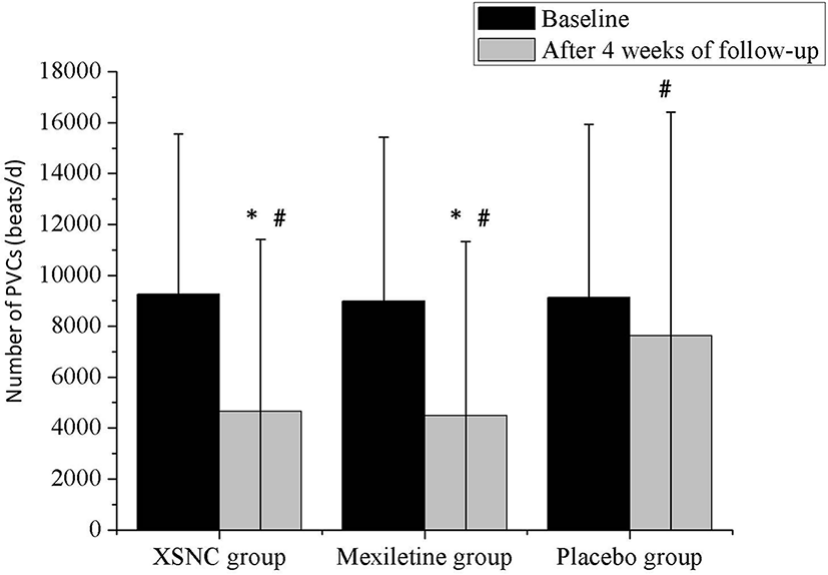
The total number of PVCs from baseline to 4 weeks after treatment in the **Xin Su Ning (**XSN) group, mexiletine group, and the placebo group. PVCs: premature ventricular contractions. ^#^P < 0.001 vs. baseline, *P < 0.001 vs. the placebo group. Continuous data were presented as the mean ± standard deviation (SD). A smaller number of PVCs was observed after a 4-week treatment than at baseline, in the XSN group (4645.89 ± 6772.17 vs. 9250.56 ± 6297.37 beats/d, P < 0.0001), mexiletine group (4480.37 ± 6851.37 vs. 8983.23 ± 6439.02 beats/d, P < 0.0001), and the placebo group (7617.16 ± 8794.66 vs. 9129.63 ± 6796.15 beats/d, P < 0.0001). In addition, compared to the placebo group, the XSN group and mexiletine group had a statistically significant change in the total PVC frequency after the 4-week treatment period (P < 0.0001).

There was no significant difference in the total number of PVCs at baseline between the XSN group, mexiletine group and the placebo group (*P* =0.5886, **Table 2**). Whereas a smaller number of PVCs was observed after a 4-week treatment than at baseline, in the XSN group (4645.89 ± 6772.17 vs. 9250.56 ± 6297.37 beats/d, *P* < 0.0001), mexiletine group (4480.37 ± 6851.37 vs. 8983.23 ± 6439.02 beats/d, *P* < 0.0001), and the placebo group (7617.16 ± 8794.66 vs. 9129.63 ± 6796.15 beats/d, *P* < 0.0001); in addition, compared to the placebo group, the XSN group and mexiletine group had a statistically significant change in the total PVC frequency after the 4-week treatment period (*P* < 0.0001, **Figure 6**). In the FAS analysis (**Table 2**), in the comparison between the pretreatment and 4 weeks post-treatment data, the SER and ER were 45.73% and 20.12% in XSN group, 40.77% and 22.32% in mexiletine group, and 14.55% and 12.73% in placebo group, respectively; the proportion of patients with a TER reduction in the frequency of total PVCs for the XSN, mexiletine, and placebo groups were 65.85%, 63.10, and 27.27%, respectively (*P* < 0.0001); the proportion of patients with a TER reduction in the frequency of PVCs was significantly greater in the XSN group compared with the placebo group (38.58%, 95% *CI*: 30.07–47.10%, *P* < 0.0001). The PPS analysis showed similar trends (39.78%, 95% *CI*: 30.07–48.68%, *P* < 0.0001, **Table 3**).

**Table 2:** Primary and secondary endpoint results after 4 weeks of follow-up in the FAS, n (%).

Items	XSN group (n=328)	Mexiletine group (n=336)	Placebo group (n=165)	Rate difference,% (95% CI)	P
Primary endpoints					
SER	150 (45.73)	137 (40.77)	24 (14.55)		
ER	66 (20.12)	75 (22.32)	21 (12.73)		
TER	216 (65.85)	212 (63.10)	45 (27.27)	38.58 (30.07–47.10)	<0.0001
Secondary endpoints					
Palpitation					
SER	163 (52.41)	137(41.03)	32 (20.65)		
ER	91 (29.26)	81 (24.85)	52 (33.55)		
TER	254 (81.67)	218 (66.88)	84 (54.2)		<0.0001
Chest discomfort					
SER	189 (60.77)	174 (53.37)	57 (36.78)		
ER	56 (18.01)	48 (14.72)	29 (18.71)		
TER	245 (78.78)	222 (68.09)	86 (55.49)		<0.0001

FAS, full analysis set; CI, confidence interval; SER, significantly effective response; ER, effective response; TER, total effective response; rate difference, rate of TER in XSN group − rate of TER in placebo group; P, XSN group vs. placebo group.

**Table 3:** Primary and secondary endpoint results after 4 weeks of follow-up in the PPS, n (%).

Items	XSN group (n=307)	Mexiletine group (n=320)	Placebo group (n=152)	Rate difference,% (95% CI)	P
Primary endpoints					
SER	148 (48.21)	137 (42.81)	24 (15.79)		
ER	65 (21.17)	75 (23.44)	21 (13.82)		
TER	213 (69.38)	212 (66.25)	45 (29.61)	39.78 (30.07–48.68)	<0.0001
Secondary endpoints					
Palpitation					
SER	161 (52.45)	135 (42.19)	31 (20.39)		
ER	90 (29.32)	79 (24.69)	52 (34.21)		
TER	251 (81.77)	214 (66.88)	83 (54.6)		<0.0001
Chest discomfort					
SER	187 (60.91)	171 (53.44)	55 (36.19)		
ER	55 (17.92)	48 (15.00)	29 (19.08)		
TER	242 (78.83)	219 (68.44)	84 (55.27)		<0.0001

PPS, per−protocol set; CI, confidence interval; SER, significantly effective response; ER, effective response; TER, total effective response; Rate difference, rate of TER in XSN group − rate of TER in placebo group; P, XSN group vs. placebo group.

In terms of the secondary endpoints, there were no significant differences in the severity of PVC-related symptoms, including palpitation and chest discomfort, at baseline between the XSN group, mexiletine group, and the placebo group (**Table 4**). Nevertheless, significant differences were observed in the respective PVC-related symptoms at the final visit between the XSN group, mexiletine group, and the placebo group (**Table 4**). When analyzed by FAS and PPS, patients receiving the XSN treatment experienced significantly greater SER, ER as well as TER of improvements in their PVC-related symptoms including palpitation and chest discomfort compared to patients receiving the placebo treatment (all P < 0.0001, **Tables 1** and **2**).

**Table 4:** A comparison of PVC-related symptom severity among XSN, mexiletine, and placebo group at baseline and 4 weeks after treatment, n (%).

Items	Baseline				4 weeks after treatment			
	XSN group (n=328)	Mexiletine group (n=336)	Placebo group (n=165)	P	XSN group (n=311)	Mexiletine group (n=326)	Placebo group (n=155)	P
Palpitation								
None	27 (8.23)	23 (6.85)	6 (3.64)	0.6171	147 (47.27)	114 (34.97)	32 (20.65)	<0.0001
Mild	97 (29.57)	118 (35.12)	57 (34.55)		145 (46.62)	177 (54.29)	93 (60.00)	
Moderate	178 (54.27)	161 (47.92)	84 (50.91)		19 (6.11)	31 (9.51)	26 (16.77)	
Severe	26 (7.93)	34 (10.12)	18 (10.91)		0 (0.00)	4 (1.23)	4 (2.58)	
Chest discomfort								
None	42 (12.80)	40 (11.90)	17 (10.30)	0.9471	187 (60.13)	171 (52.45)	56 (36.13)	<0.0001
Mild	146 (44.51)	163 (48.51)	83 (50.30)		118 (37.94)	134 (41.10)	82 (52.90)	
Moderate	136 (41.46)	122 (36.31)	62 (37.58)		6 (1.93)	21 (6.44)	15 (9.68)	
Severe	4 (1.22)	11 (3.27)	3 (1.82)		0 (0.00)	0 (0.00)	2 (1.29)	

PVC, premature ventricular contraction; P, XSN group vs. placebo group; XSN, Xin Su Ning.

#### Safety Analysis

The adverse events are presented in **Table 5**. A total of 861 patients were included in the SS analysis. Adverse events were reported in 50 patients, including 18 in the XSN group, 21 in the mexiletine group, and 11 in the placebo group (P = 0.8430). All reported adverse events were mild and were judged to be probably not attributable to XSN. Neither death nor serious adverse events related to XSN was reported during the study. Compared with the placebo group, the administration of XSN had no significant impact on the liver, kidney functions as well as the ECG parameters (**Table 6**).

**Table 5:** Adverse events reported during the study, n (%).

Items	XSN group (n=328)	Mexiletine group (n=336)	Placebo group (n=165)
Headache	2	2	1
Dizziness	3	3	2
Palpitation	0	1	0
Sinus bradycardia	0	0	0
Atriaventricular block	0	0	0
Gastrointestinal symptoms	3	3	2
Abnormal liver function	2	3	2
Urinary infection	1	2	0
Anemia	1	1	0
Rash	2	3	2
Conjunctivitis	2	2	1
Osphyalgia	2	1	1
Total adverse events	18 (5.25)	21 (6.09)	11 (6.36)

XSN, Xin Su Ning.

**Table 6:** Changes in the laboratory tests and ECG parameters between the two groups before and after treatment, medium (range).

Variables	XSN group		Mexiletine group		Placebo group	
	0 weeks	4 weeks	0 weeks	4 weeks	0 weeks	4 weeks
Laboratory tests						
ALT, IU/L	33 (4–41)	21 (5–40)	38 (9–50)	32 (7–48)	21 (8–42)	22 (8–43)
AST, IU/L	27 (4–40)	18 (21–72)	21 (5–40)	18 (17–59)	20 (6–45)	23 (7–53)
Scr, mmol/L	71 (62–106)	69 (60–106)	60 (53–97)	61 (44–105)	51 (44–104)	56 (44–106)
ECG parameters						
QT interval, ms	402 (280–464)	399.5 (297–442)	405 (297–493)	406 (318–483)	397 (326–492)	402 (337–487)
Mean HR, bpm	73 (67–103)	73 (67–96)	74 (67–89)	73 (68–89)	72 (67–99)	72 (64–93)
Maximum, HR, bpm	114 (102–127)	115 (101–127)	114 (103–128)	114 (102–126)	112 (100–124)	114 (102–126)
Minimum, HR, bpm	51 (47–75)	51 (46–86)	51 (47–75)	52 (47–71)	52 (36–78)	51 (36–75)

ALT, alanine aminotransferase; AST, aspartate aminotransferase; Scr, serum creatinine; ECG, electrocardiogram; HR, heart rate; XSN, Xin Su Ning.

## Discussion

As a TCM formula, XSN has been certified to treat tachycardia type of arrhythmias since 2005. Its clinical efficacy has been widely accepted. The results we reported here provided further and rigorous cellular mechanistic and clinical evidences helping to understand why and how XSN exert its antiarrhythmic actions clinically in its everyday applications.

Previously we reported that XSN blocks human sodium and potassium channels (Wang et al., 2019b). We report here that XSN prolongs APD in a dose-dependent and reversible manner. These pharmacological findings have clearly classified XSN as a class I & III antiarrhythmic medicine (Vaughan Williams, 1992). These cellular pharmacological evidence, therefore, provides support for XSN’s clinical antiarrhythmic applications, which is further supported by the clinical trial data we presented here.

Our clinical data describe the first three-armed, randomized, double-blind, placebo-controlled, parallel-group, multicenter trial regarding TCM treatment for PVCs. Although conventional AADs are the first-line therapy for patients with incapacitating PVC-related symptoms, due to the proarrhythmic effects and adverse side effect of most AADs, the clinical use of these drugs has been severely restricted. In our study, we found that XSN improved the overall counts of PVCs and PVC-related symptoms in a pre- and post-treatment analysis, and its efficacy was noninferior to that of mexiletine, a class Ib conventional AAD and was significantly superior to that of the placebo. In addition, the results of the present study also demonstrated that XSN was tolerated and safe in patients with PVCs during the trial treatment, which support the finding that there has no severe adverse reactions induced by XSN being reported since 2005 when it was certified to be sold in China.

Some limitations in our study must be acknowledged. Firstly, for the experimental data, although the antiarrhythmic effect of XSN in animal experiments was presented for the certification to produce in 2005 in China (unpublished), the report we presented here does not include the effect of XSN in arrhythmic animal models, we, therefore, aim in the near future to carry out experiments that should reflect the clinical efficacy of XSN in animal models to support our cellular electrophysiological findings. Secondly, for the clinical study, the follow-up period of our study was only 4 weeks and this period may be short and not sufficient to evaluate the medium- to long-term effects of XSN for the treatment of PVCs, as well as the adverse events associated with long-term therapy. Long-term clinical trials are needed to further verify the results of our study and assess the long-term efficacy and safety of XSN in the treatment of PVCs. Secondly, not all patients enrolled in the current study have structural heart disease. However, in the real world, patients with PVCs are often suffered from organic heart diseases, such as coronary heart disease, hypertension, primary cardiomyopathy, or heart failure. These patients should be included and analyzed in future clinical trials.

XSN formula was designed to treat arrhythmia with phlegm-heat heart-disturbed syndrome (PHHD) according to Chinese medicine theory. We recently reported a network/systematic pharmacology study on XSN with a novel weight coefficient introduced to mimic the relative amount of all the components in relation with the weight of the corresponding herb in the formula (Wang et al., 2019a). Combining the collected data and our discoveries in the lab, a panoramagram of the pharmacological mechanism of XSN was established. Pathway enrichment and analysis showed that XSN treated PHHD arrhythmia through multiple ion channels regulation, protecting the heart from I/R injury, inhibiting the apoptosis of cardiomyocyte, and improving glucose and lipid metabolism. This study demonstrated the wider spectrum of the likely pharmacological effect of XSN based on its multi-targeting property that support the clinical therapeutic outcome observed in nearly 15 years of its clinical use.

With the support of our previous discoveries (Wang et al., 2019a; Wang et al., 2019b) and data presented here, we can conclude that the antiarrhythmic action of XSN does have cellular electrophysiological basis with class I & III antiarrhythmic characteristics (Vaughan Williams, 1992). In addition, the widely accepted computational simulation of human APs based on our experimental data with XSN has also shown the class III antiarrhythmic characteristics of prolonging APD, an action that increases the effective refractory period which suppresses tachyarrhythmia, PVCs, caused by reentry mechanisms. XSN blocks the whole-cell I_K_ would contribute to the APD prolongation. The difference on APD prolongation between XSN and liensinine indicate that one single compound does not assemble XSN’s action on APD. The clinical study showed significant inhibition of PVCs in patients, which can be mechanistically supported by our cellular electrophysiological discoveries.

## Data Availability Statement

All datasets generated for this study are included in the article/**Supplementary Material**.

## Ethics Statement

The studies involving human participants were reviewed and approved by the Institutional Review Boards of each participating center in China, and the study was conducted in accordance with the principles of the Good Clinical Practice Guidelines and the Declaration of Helsinki. The patients/participants provided their written informed consent to participate in this study. The study was registered in the Chinese Clinical Trial Register Center (ChiCTR-TRC-14004180). The animal study was carried out in accordance with UK Home Office regulations (Schedule I of A [SP] A 1986), approved by national and University ethics committees.

## Author Contributions

Y-LM conceived, designed, and conducted the cellular electrophysiological study. TW and PN participated the cellular experiment and computational modeling study. XY designed the clinical study. RW, CE, CC, and DN helped with lab support, read the paper and gave critical suggestions of changes of the paper. XY, R-MH, JY, WL, JZ, HH, XG, MC, YW, MW, JM, XM, LQ, HW, FL, YC, SG, and WG conducted the clinical trial study. YM, R-MH, and XY wrote the paper.

## Funding

The cellular work was supported by grants from the Chinese Medicine Research Fund, University of Oxford. The grant was funded by Shaanxi Momentum Pharmaceutical Co., Ltd. Shaanxi Momentum Pharmaceutical Co., Ltd. had no involvement in the study design, data collection and analysis, decision to publish, or preparation of the manuscript. The clinical study was supported by the National Natural Science Foundation of China (81970271 to XY), Beijing Natural Science Foundation (7172080 to XY), National Natural Science Foundation (81800293 to R-MH), and Beijing Hospitals Authority Youth Programme (QML2019305 to R-MH).

## Conflict of Interest

The authors declare that the research was conducted in the absence of any commercial or financial relationships that could be construed as a potential conflict of interest.

The reviewer JC declared a shared affiliation with several of the authors, WL, YU, to the handling editor at the time of the review.

## References

[B1] Adams J. C. Srivathsan K. Shen W. K. (2012). Advances in management of premature ventricular contractions. J. Interv Card Electrophysiol 35 (2), 137–149. 10.1007/s10840-012-9698-x 22875587

[B2] Ataklte F. Erqou S. Laukkanen J. Kaptoge S. (2013). Meta-analysis of ventricular premature complexes and their relation to cardiac mortality in general populations. Am. J. Cardiol. 112 (8), 1263–1270. 10.1016/j.amjcard.2013.05.065 23927786

[B3] Brenyo A. Aktas M. K. (2014). Review of complementary and alternative medical treatment of arrhythmias. Am. J. Cardiol. 113 (5), 897–903. 10.1016/j.amjcard.2013.11.044 24528618

[B4] Cantillon D. J. (2013). Evaluation and management of premature ventricular complexes. Cleve Clin. J. Med. 80 (6), 377–387. 10.3949/ccjm.80a.12168 23733905

[B5] Eugenio P. L. (2015). Frequent Premature Ventricular Contractions: An Electrical Link to Cardiomyopathy. Cardiol. Rev. 23 (4), 168–172. 10.1097/CRD.0000000000000063 25741605

[B6] Frolkis J. P. Pothier C. E. Blackstone E. H. Lauer M. S. (2003). Frequent ventricular ectopy after exercise as a predictor of death. N Engl. J. Med. 348 (9), 781–790. 10.1056/NEJMoa022353 12606732

[B7] Frommeyer G. Eckardt L. (2016). Drug-induced proarrhythmia: risk factors and electrophysiological mechanisms. Nat. Rev. Cardiol. 13 (1), 36–47. 10.1038/nrcardio.2015.110 26194552

[B8] Giles K. Green M. S. (2013). Workup and management of patients with frequent premature ventricular contractions. Can. J. Cardiol. 29 (11), 1512–1515. 10.1016/j.cjca.2013.08.005 24182757

[B9] Guo R. Zhang X. Su J. Xu H. Zhang Y. Zhang F. (2018). Identifying potential quality markers of Xin-Su-Ning capsules acting on arrhythmia by integrating UHPLC-LTQ-Orbitrap, ADME prediction and network target analysis. Phytomedicine: 44, 117–128. 10.1016/j.phymed.2018.01.019 29526583

[B10] Hua W. Gao R. L. Zhao B. C. Wang J. Chen X. H. Cai C. (2015). The Efficacy and Safety of Wenxin Keli in Patients with Frequent Premature Ventricular Contractions: A Randomized, Double-blind, Placebo-controlled, Parallel-group, Multicenter Trial. Chin Med. J. (Engl) 128 (19), 2557–2564. 10.4103/0366-6999.166026 26415790 PMC4736861

[B11] Kowey P. R. Yan G. X. (2005). Proarrhythmias and antiarrhythmias: two sides of the same coin. Heart Rhythm. 2 (9), 957–959. 10.1016/j.hrthm.2005.06.003 16171750

[B12] Lee C. H. Park K. H. Nam J. H. Lee J. Choi Y. J. Kong E. J. (2015). Increased variability of the coupling interval of premature ventricular contractions as a predictor of cardiac mortality in patients with left ventricular dysfunction. Circ. J. 79 (11), 2360–2366. 10.1253/circj.CJ-15-0732 26356836

[B13] Liu W. Xiong X. Feng B. Yuan R. Chu F. Liu H. (2015). Classic herbal formula Zhigancao Decoction for the treatment of premature ventricular contractions (PVCs): a systematic review of randomized controlled trials. Complement Ther. Med. 23 (1), 100–115. 10.1016/j.ctim.2014.12.008 25637158

[B14] Ma Y. L. Bates S. Gurney A. M. (2006). The effects of paeonol on the electrophysiological properties of cardiac ventricular myocytes. Eur. J. Pharmacol. 545 (2-3), 87–92. 10.1016/j.ejphar.2006.06.064 16876781

[B15] Qureshi W. Shah A. J. Salahuddin T. Soliman E. Z. (2014). Long-term mortality risk in individuals with atrial or ventricular premature complexes (results from the Third National Health and Nutrition Examination Survey). Am. J. Cardiol. 114 (1), 59–64. 10.1016/j.amjcard.2014.04.005 24819898 PMC4334655

[B16] Rosen M. R. (1988). Mechanisms for arrhythmias. Am. J. Cardiol. 61 (2), 2a–8a. 10.1016/0002-9149(88)90735-7 3276123

[B17] Saurav A. Smer A. Abuzaid A. Bansal O. Abuissa H. (2015). Premature ventricular contraction-induced cardiomyopathy. Clin. Cardiol. 38 (4), 251–258. 10.1002/clc.22371 25678299 PMC6711062

[B18] Vaughan Williams E. M. (1992). Classifying antiarrhythmic actions: by facts or speculation. J. Clin. Pharmacol. 32 (11), 964–977. 10.1002/j.1552-4604.1992.tb03797.x 1474169

[B19] Wang T. Streeter H. Wang X. Purnama U. Lyu M. Carr C. (2019a). A Network Pharmacology Study of the Multi-Targeting Profile of an Antiarrhythmic Chinese Medicine Xin Su Ning. Front. Pharmacol. 10, 1138. 10.3389/fphar.2019.01138 31607935 PMC6774044

[B20] Wang T. Xie W. Yu J. Ellory C. Wilkins R. Zhu Y. (2019b). Ion Channel Targeted Mechanisms of Anti-arrhythmic Chinese Herbal Medicine Xin Su Ning. Front. Pharmacol. 10, 70. 10.3389/fphar.2019.00070 30787875 PMC6372541

[B21] Weiss J. N. Garfinkel A. Karagueuzian H. S. Nguyen T. P. Olcese R. Chen P. S. (2015). Perspective: a dynamics-based classification of ventricular arrhythmias. J. Mol. Cell Cardiol. 82, 136–152. 10.1016/j.yjmcc.2015.02.017 25769672 PMC4405495

[B22] Winkens R. A. Hoppener P. F. Kragten J. A. Verburg M. P. Crebolder H. F. (2014). Are premature ventricular contractions always harmless? Eur. J. Gen. Pract. 20 (2), 134–138. 10.3109/13814788.2013.859243 24286118

[B23] Xiong X. Yang X. Liu Y. Zhang Y. Wang P. Wang J. (2013). Chinese herbal formulas for treating hypertension in traditional Chinese medicine: perspective of modern science. Hypertens. Res. 36 (7), 570–579. 10.1038/hr.2013.18 23552514 PMC3703711

