# Supplementary figures and images for: Investigation of the Cellular Pharmacological Mechanism and Clinical Evidence of the Multi-Herbal Antiarrhythmic Chinese Medicine Xin Su Ning
Source: Front Pharmacol. 2020 May 6;11:600. doi: 10.3389/fphar.2020.00600 (PMC7218142; doi:10.3389/fphar.2020.00600)

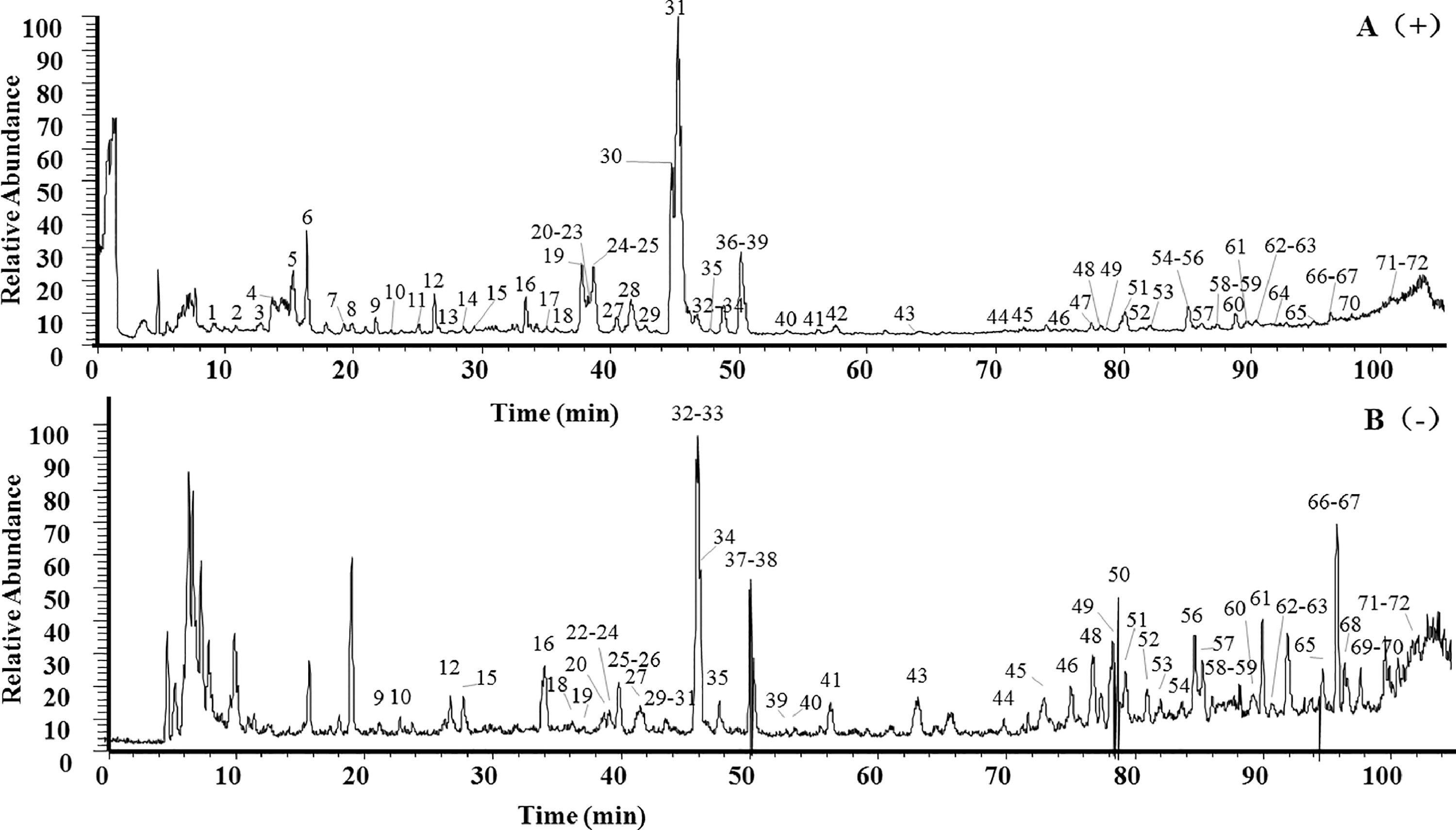

Supplement: Supplementary file 2 [file Image_1.jpeg]
